# Superior sensation: superior colliculus participation in rat vibrissa system

**DOI:** 10.1186/1471-2202-8-12

**Published:** 2007-01-31

**Authors:** Marie E Hemelt, Asaf Keller

**Affiliations:** 1 Program in Neuroscience, University of Maryland School of Medicine, Baltimore, MD, USA; 2 Department of Anatomy & Neurobiology, University of Maryland School of Medicine, Baltimore, MD, USA

## Abstract

**Background:**

The superior colliculus, usually considered a visuomotor structure, is anatomically positioned to perform sensorimotor transformations in other modalities. While there is evidence for its potential participation in sensorimotor loops of the rodent vibrissa system, little is known about its functional role in vibrissa sensation or movement. In anesthetized rats, we characterized extracellularly recorded responses of collicular neurons to different types of vibrissa stimuli.

**Results:**

Collicular neurons had large receptive fields (median = 14.5 vibrissae). Single units displayed responses with short latencies (5.6 ± 0.2 msec, median = 5.5) and relatively large magnitudes (1.2 ± 0.1 spikes/stimulus, median = 1.2). Individual neurons could entrain to repetitive vibrissa stimuli delivered at ≤ 20 Hz, with little reduction in phase locking, even when response magnitude was decreased. Neurons responded preferentially to vibrissa deflections at particular angles, with 43% of the cells having high (≥ 5) angular selectivity indices.

**Conclusion:**

Results are consistent with a proposed role of the colliculus in somatosensory-mediated orienting. These properties, together with the connections of the superior colliculus in sensorimotor loops, are consistent with its involvement in orienting, alerting and attentive functions related to the vibrissa system.

## Background

The superior colliculus has long been studied as a center for visual sensory and motor responses [1-3], and is involved in orienting attention [4-6] and multimodal processing [7-10]. The functions of the colliculus have been characterized principally in relation to vision and eye movements, whereas its role in other sensorimotor transformations has received less attention. We seek to explore the function of the superior colliculus using the vibrissa system, a vital sensorimotor system for the rat. Although previous studies demonstrated the existence of vibrissa-responsive collicular neurons [9,11-13], there have been no systematic, quantitative analyses of their response properties to controlled stimuli. The goal of this study was to correct this deficiency.

The superior colliculus is thought to be part of a sensorimotor loop in the rat vibrissa system, receiving input from trigeminal nuclei and projecting to the vibrissa motoneurons [see [14]]. In addition, the superior colliculus interacts with other structures of the vibrissa-barrel system: it receives input from the barrel cortex and from the vibrissa area of the motor cortex [15-17], and receives inputs from the cerebellum and basal ganglia [see [18]]. It is reciprocally connected with the zona incerta [19], a structure recently shown to be involved in state dependent gating of vibrissal inputs in the thalamus [20-22]. Because of these anatomical relationships, and because of their role in orienting responses, we hypothesized that collicular neurons respond with short latency and strong directional selectivity to vibrissal stimuli.

## Results

### Location of vibrissa responsive units

We report data from 51 vibrissa-responsive units recorded from 24 animals. We used histological analyses, stereotaxic coordinates and depth readings from the manipulator to determine the location of the recorded units. Due to the size of the lesions, we did not separate intermediate gray and white layers, but divided the colliculus into superficial, intermediate, and deep layers. Vibrissa responsive units were located in the intermediate layers of the lateral and posterior parts of the colliculus. Stereotaxic coordinates were 2.2 to 2.6 mm lateral and 7.6 to 7.9 mm posterior to Bregma, at a depth of 3.2 to 5.3 below the cortical surface. Clusters of vibrissa responsive neurons were interspersed with clusters responding to manual stimulation of other body parts. Vibrissa-responsive clusters ranged in size from 100 to 300 microns along the axis of penetration; it was not possible to reliably determine the cluster size in the horizontal plane without fine-grained mapping in a single animal. The clustering of vibrissa responses may reflect the clustering in the colliculus of afferents from the trigeminal nuclei [23,24].

### Waveform morphology

**Figure 1:**
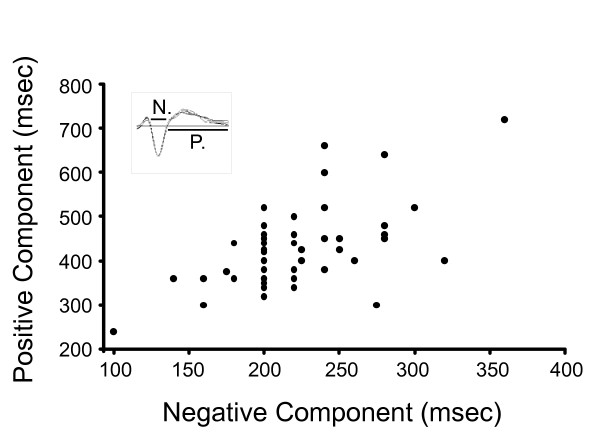
**Neuronal subclasses could not be distinguished by their waveform morphology**. The inset shows representative waveforms, with the negative (N) and positive (P) components indicated. When the positive component was plotted against the negative component, data points formed a single cluster.

Neurons in several brain regions have been classified according to their action potential waveforms [25,26]. For example, cortical vibrissa-responsive units include regular and fast spiking neurons [27,28]. In extracellular recordings, inhibitory, fast spiking neurons have action potentials with a brief (≤ 180 μsec) negativity followed by a brief positivity (≤ 400 μsec), whereas regular spiking neurons have longer waveform components (> 180 μsec and > 400 μsec). The superior colliculus contains both excitatory (glutamatergic) and inhibitory (GABAergic) neurons, which may segregate into similar categories [18,29]. To test this, we measured the two components of their extracellularly recorded waveforms, and plotted the positive component against the negative component (Fig. 1). All data points (51 data points, 12 overlapping) formed a single cluster with values encompassing those of cortical regular and fast spiking units. While we cannot exclude the possibility that our sample included a heterogeneous population of neurons, these findings suggest that our recordings could not distinguish neuronal subclasses based on their waveforms.

### Spontaneous activity

**Figure 2:**
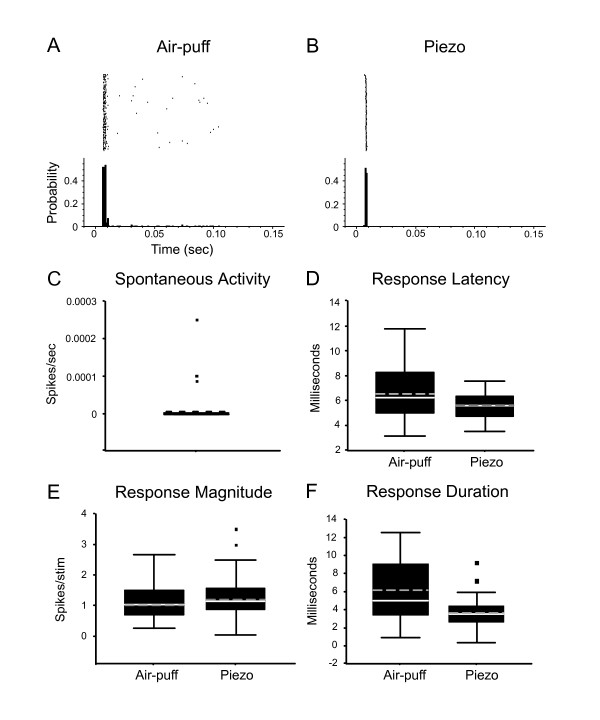
**Response kinetics of superior colliculus neurons**. *A-C: *PSTHs of representative responses constructed with 1 msec bins. Collicular responses to air-puffs (*A*) or piezoelectric (*B*, piezo) vibrissa deflections. *C-F: *Boxplots depict median (solid line), mean (dotted line) and 1^st ^and 3^rd ^quartiles (box). Whiskers represent data within 1.5 times the range from the 1^st ^to the 3^rd ^quartile. Squares represent outliers (data outside 1.5 times the range from the 1^st ^to the 3^rd ^quartile). *C: *Neurons in the superior colliculus have low or no spontaneous activity. *D: *Response latencies evoked by air-puff deflections were not significantly different than those evoked by piezoelectric deflections (*P *= 0.2). *E: *Response magnitude was unaffected by stimulus type *(P *= 0.7). *F*: Response durations were short and unaffected by stimulus type (*P *= 0.07). All statistical comparisons use the Kolmogorov-Smirnov test.

Vibrissa-responsive neurons had low or no spontaneous activity (e.g. Fig. 2A–C; 44/48 neurons had *no *spontaneous activity, mean was 0.01 ± 0.006 Hz). Therefore, to avoid biasing our recording to spontaneously active units, we searched for units while manually stimulating the vibrissae. We did find many spontaneously active, albeit non-responsive units near these relatively quiescent, responsive neurons. We found no neurons for which stimulation evoked a decrease in spontaneous activity.

### Response latency

The superior colliculus receives input from a variety of structures that process vibrissa inputs, including the trigeminal nuclear complex and the neocortex (see Background). We reasoned that if collicular responses represent direct inputs from the trigeminal nuclei, these responses would have relatively short latencies, similar to those of thalamic neurons that receive trigeminal inputs. Figures 2A–B depict responses recorded from two representative collicular neurons, showing the latency of their responses to vibrissa stimuli delivered as an air-puff (Fig. 2A; 6.4 msec onset latency) or delivered with the piezoelectric device (Fig. 2B; 7.0 msec). For consistency, here and in subsequent analyses (magnitude, duration, and response ratios), we report responses to the vibrissa deflections in the caudal direction, the direction mimicking most vibrissa contacts during active whisking. When using the piezoelectric device, we stimulated the vibrissa evoking the largest response.

Group data are depicted in the boxplots in Figure 2D. Piezoelectric stimulation of a single vibrissa elicited a response with median latency of 5.5 msec (5.6 ± 0.2 msec, n = 31). In response to air-puff stimulation of multiple vibrissae, the median latency was 6.2 msec (6.5 ± 0.6, n = 15). There is no significant difference in latency between these two groups (*P *= 0.2). Thus, collicular neurons display short latency responses to vibrissa inputs, similar to their counterparts in the ventrobasal thalamus.

### Response kinetics

**Figure 3:**
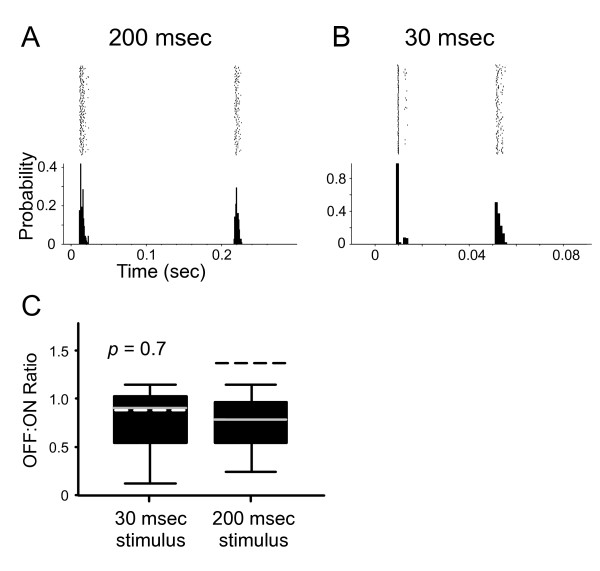
**OFF:ON response magnitude ratios for vibrissa-responsive neurons**. Examples of responses to 200 msec stimuli (A) and 30 msec stimuli (B) in different neurons. C: Boxplots depicting OFF:ON ratios for 200 and 30 msec stimuli. Decreasing the stimulus duration had little effect on the OFF:ON ratio (P = 0.7).

Ramp-and-hold stimuli, delivered with the piezoelectric device, evoked phasic responses in all 31 neurons tested, with robust spiking only at the onset (ON) and offset (OFF) of the stimuli (Fig. 3A, 200 msec stimuli; Fig. 3B, 30 msec stimuli). The durations of ON responses evoked with piezoelectric deflections were very short, with all 31 neurons showing durations less than 10 msec (26 less than 5 msec, 3.7 ± 0.4, Fig. 2F). Air-puff stimuli produced more temporally dispersed responses (e.g., Fig. 2A), and, as a result, response duration was more varied but not significantly different from the piezoelectric evoked responses (range 1.0–12.6 msec, 6.2 ± 0.92 msec, n = 16, *P *= 0.07). Using criteria introduced by Simons and Carvell [30] we classified all the neurons tested as rapidly adapting (responses lasting < 50 msec).

Mean response magnitudes to caudal vibrissa deflections (spikes per stimulus, see Methods) were 1.1 ± 0.1 for air-puff stimulation (n = 16), and 1.2 ± 0.1 for piezoelectric stimulation (n = 31, Fig. 2E). We also tested the response to stimuli in the preferred direction (the direction evoking the largest response); piezoelectric stimulation produced a mean response magnitude of 1.6 ± 0.2 (median 1.3, n = 23). The angular selectivity of these neurons is described in detail below.

In both cortex and thalamus, OFF response magnitudes are generally smaller than ON response magnitudes [31]. When we applied a 200 msec caudal deflection with the piezoelectric device, the OFF:ON ratio was 1.4 ± 0.5 (0.5 n = 20 where deflection evoked both a detectable ON and OFF response), larger than that reported previously in barrel cortex [0.55 ± 0.03, [31]]. When the stimulus duration was decreased to 30 msec (*e.g. *Fig. 3B), the OFF response decreased somewhat, though the evoked OFF:ON ratio was statistically indistinguishable from that of the longer deflection (Fig. 3C, ratio = 0.9 ± 0.1, *P *= 0.3, n = 13, unpaired t-test). Thus, changing stimulus duration had no significant effect on OFF:ON response magnitude ratios.

### Receptive field size

**Figure 4:**
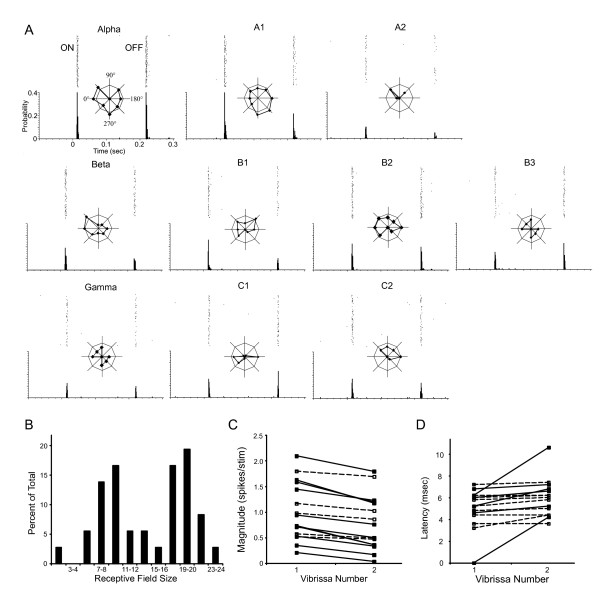
**Superior colliculus neurons have large receptive fields with principal whiskers**. *A*: An example of a neuron with a 17-vibrissa receptive field. Responses to individual deflections of 10 vibrissae with the piezoelectric device are depicted, with the vibrissa deflected indicated above each PSTH. Vibrissae were deflected in eight different directions (0° is caudal, 90° is dorsal), and ON responses to each direction were used to create the polar tuning curves. Distance from the center of the polar tuning curve represents the magnitude of the response for deflections in that direction. The radii of the octagons represent one spike/stimulus. The PSTHs were constructed by pooling spikes recorded in response to each of the eight directions. *B: *Histogram of receptive field sizes of collicular neurons, defined as the number of vibrissae evoking responses to manual stimulation. *C: *Difference in response magnitude to stimulation of different vibrissae. For each neuron, vibrissae were ordered according to their response magnitudes ("V1" evoked the largest magnitude response, "V2" the second largest magnitude, etc). Eleven of 16 neurons showed a significant difference in magnitude between V1 and V2 (solid lines, paired t-test, P < 0.005). *D*: Difference in onset latency to stimulation of different vibrissae. For each neuron, vibrissae were ordered according to their response latency ("V1" evoked responses at the shortest latency, etc). Six of 16 neurons showed a significant difference in latency between V1 and V2 (solid lines, paired t-test, P < 0.005).

Receptive field size varies along the vibrissa-barrel neural axis, from a single vibrissa in the trigeminal ganglion to a median of nine vibrissae in SII cortex [28,32]. We tested the receptive field of 36 neurons by manually deflecting individual vibrissae, and monitoring the responses on an oscilloscope and through an audio amplifier. Figure 4B shows the distribution of receptive field sizes for collicular neurons (median = 14.5 vibrissae, 13.8 ± 0.1). Vibrissae encompassing the receptive field of a particular neuron were always located contiguously on the whisker pad (e.g. Fig 4A). These large receptive fields are compared below to others in the vibrissa-barrel axis.

### Principal vibrissa analysis

In the barrel cortex, neurons responding to stimulation of more than one vibrissa respond preferentially to the vibrissa associated with their anatomically defined barrel. This vibrissa is referred to as the principal whisker, and its stimulation evokes a response with larger magnitude and shorter onset latency than stimulation of other vibrissae in the receptive field [26,28]. We examined both response magnitude and latency as potential metrics for distinguishing principal whiskers in the superior colliculus.

We examined responses to individual vibrissae within a receptive field using piezoelectric deflections. In cases where unit isolation degraded before the experiment was completed, it was not possible to test all the vibrissae associated with a particular neuron. Because of the smaller amplitude of vibrissa deflections produced with the piezoelectric device, not all the vibrissae within the receptive field–defined with manual stimulation–produced significant responses to piezoelectric stimuli.

Figure 4A shows responses of a single neuron to deflections of 10 different vibrissae within a 17-vibrissa receptive field (A3, B4, C3, C4, C5, γ and D5 were not tested with piezoelectric stimulation). Each vibrissa was deflected at eight different angles, pseudo-randomly, with the piezoelectric device. In these polar plots, the distance from the origin represents the magnitude of the ON response for deflections at that angle; the angular selectivity of this neuron is discussed below. The overall response of the neuron to each vibrissa was obtained by pooling responses to all eight directions and constructing PSTHs, shown here below the polar plots. Because different whiskers within a receptive field did not have the same preferred direction, we used these PSTHs, which contain information from all eight deflection angles, for the analysis of response magnitude and latency as metrics for determining a principle whisker. The representative neuron in Figure 4A responded to each of the vibrissae with similar magnitude and latency.

This analysis was performed on 16 neurons for which we tested at least 5 vibrissae. For each neuron, we rank-ordered the vibrissae according to the magnitude of evoked responses. The vibrissa evoking the largest response magnitude was defined as V1, the next largest as V2, etc. We compared response magnitudes for V1 and V2 using a t-test. These response magnitudes were significantly different in 69% of neurons (11 of 16, P < 0.005) indicating that deflection of this vibrissa evoked a larger magnitude response than any other vibrissa in the receptive field.

In addition, we rank-ordered these vibrissae according to the latency of evoked responses (the shortest latency being V1, etc). Although the response latencies increase somewhat (Fig 4D), we found only 6 of 16 neurons (38%) where the increase was significant between the V1 and V2. This indicates that V1 did not always evoke a response significantly faster than the vibrissa evoking responses at the next shortest latency. In eight of 16 neurons, the vibrissa evoking the largest magnitude response was also the vibrissa with the shortest latency.

Thus, collicular neurons respond to multiple vibrissae with similar latency but with different response magnitudes. This indicates that superior colliculus neurons have a principal whisker, despite their large receptive fields. Responses to non-principal (adjacent) vibrissae continue to be robust: For example, the vibrissae ranked V5 evoked a response of 0.4 ± 0.1 spikes/stimulus (n = 14), a value that was 44% of the principal whisker response. For consistency, in other analyses we focused on the vibrissa evoking the largest response, that is, the principal whisker.

### Angular tuning characteristics

**Figure 5:**
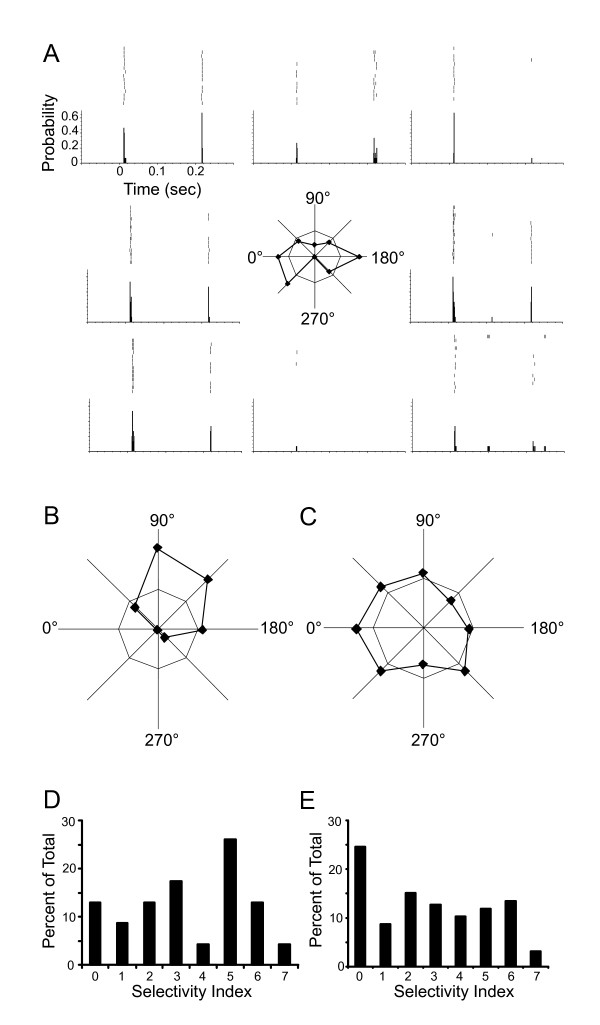
**Superior colliculus neurons display a high degree of angular selectivity**. *A: *To determine angular selectivity, vibrissae were deflected in eight directions. ON responses in each direction were used to compute the selectivity index (SI) and create the polar tuning curve (center). Distance from the center in the polar tuning curve represents the magnitude of the response for deflections in that direction. Octagon radii represent one spike/stimulus. *B: *A well-tuned neuron (SI = 5). *C: *A poorly tuned neuron (SI = 1). Histograms in *D *and *E *depict the SIs for all neurons tested, either considering only responses to the vibrissa evoking the largest response in each neuron (*D*); or considering all vibrissa-neuron pairs (*E*).

Neurons throughout the vibrissa-to-cortex pathway of the rat are selective for the angle of vibrissa deflection [26,28,30,33,34], eliciting larger magnitude responses to preferred angle deflections. The strength of neuronal angular preference varies along this neuroaxis; for example, thalamocortical neurons have stronger angular selectivity than barrel neurons [30]. To test the angular preference of collicular neurons, we deflected vibrissae in eight different directions pseudo-randomly, for a total of 120 deflections (15 in each direction, e.g. Fig. 5A). Figure 5B presents a polar tuning curve from a neuron with strong angular preference, and Figure 5C depicts results from a poorly tuned neuron. In these tuning curves, the distance from the origin represents the magnitude of the ON response to a deflection at that angle, with 0° representing the caudal direction and 90° representing the dorsal direction.

To quantify angular preference we determined, for each neuron-vibrissa pair, the number of deflection angles evoking an ON response magnitude that was statistically smaller than the response to the maximally activating angle (compared using Student's t-test, *P *< 0.005). We then categorized tuning curves into eight groups (0 to 7) representing the number of angles with significantly smaller responses. Category 0 represents the least-tuned responses (equal response to all deflection angles) and category 7 represents the best-tuned responses (respond preferentially to one deflection angle). Figure 5D shows the distribution of selectivity indices for 23 neurons, computed from responses to the vibrissa evoking the largest magnitude response. The mean selectivity index was 3.4, with well-tuned cells (categories 5 to 7) constituting 43% of these neuron-vibrissa pairs.

When we included in this analysis responses to all vibrissae (and not only to the vibrissa maximally activating the neuron), the distribution of selectivity indices shifted to less-tuned values (29% well-tuned, 126 vibrissa-neurons pairs, Fig 5E). This is because these vibrissae evoked smaller responses, many of which were statistically indistinguishable from zero. As a result, the contrast between responses to deflections at different angles was reduced. We therefore applied a second metric, the angular tuning ratio [35], computed by dividing the response magnitude in the preferred direction by the overall response magnitude. Although this metric does not allow for statistical comparisons, it is less sensitive to zero responses in a particular direction. This ratio was 1.7 ± 0.1 (median = 1.5) for vibrissa producing the largest response in each neuron, and 2.2 ± 0.1 (median = 1.7) for all vibrissa-neuron pairs. Thus, both the selectivity index and tuning ratio metrics indicate that collicular neurons respond preferentially to vibrissa deflections at particular angles.

### Response adaptation

**Figure 6:**
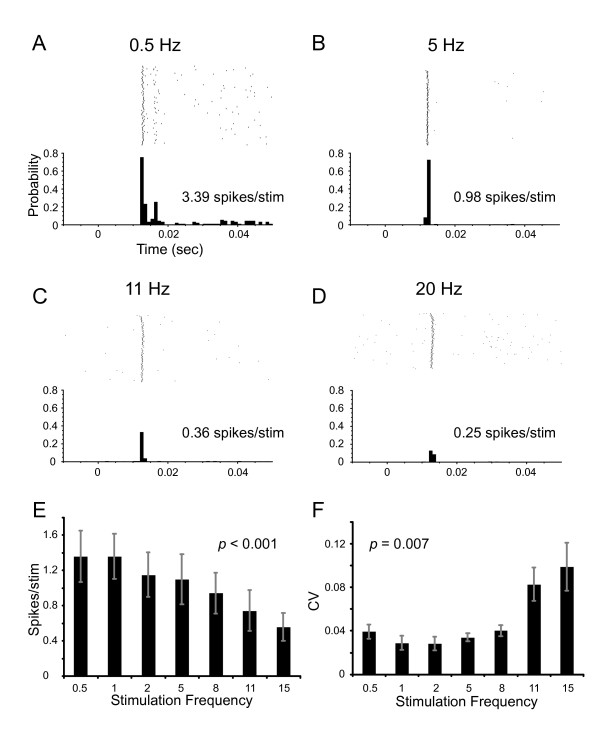
**Response adaptation in the superior colliculus**. PSTHs (*A-D*) show response characteristics of a single cell over a range of stimulation frequencies. Significant response magnitudes are indicated for each PSTH. *E: *Response magnitude decreases significantly as stimulation frequency increases (Friedman's test, *P *< 0.001). F: Phase-locking, measured as the coefficient of variation (CV) of the latency, also decreases significantly with increasing frequency stimulation (P = 0.007), but the CV remains relatively low even at high frequency.

We tested the ability of collicular neurons to respond to a range of stimulation frequencies (0.5 to 20 Hz, e.g. Fig. 6A–D). As stimulation frequency increased, response magnitude decreased for both air-puff and piezoelectric stimulation (Fig. 6E; air-puff: 1, 2, 5, 8, 11, and 20 Hz, P = 0.028, n = 10; piezoelectric: 0.5, 1, 2, 5, 8, 11, and 15 Hz, P < 0.001, n = 11; Friedman test). There were no significant differences between magnitudes of responses to piezoelectric or air-puff stimulation at any stimulation frequency (P > 1.3, 20 Hz air-puff was compared to 15 Hz piezoelectric). Response magnitude at the highest frequencies tested (15 Hz or 20 Hz) was 0.2 ± 0.07 spikes/stimulus for air-puff stimulation, and 0.6 ± 0.2 for piezoelectric stimulation.

Even when response magnitudes are reduced, phase locking of a response to a stimulus can be important in transmitting information. To test phase locking of collicular neurons at different frequencies, we computed the coefficient of variation (CV) of response latencies, using only the first spike of each response. Because the piezoelectric device generates the most temporally accurate stimulation onset, we analyze only responses to this device. The average CV for 0.5 Hz stimulation was 0.04 ± 0.006. The CV decreased significantly as stimulation frequency increased (Fig. 6F, *P *= 0.007, Friedman's test). The mean CV did not exceed 0.1 (± 0.02), a value reached at the highest stimulus frequency (15 Hz) capable of producing linear responses from the piezoelectric device. These findings indicate that, even in response to high frequency stimulation, when response magnitude is decreased, the entrainment of collicular neurons is still appreciable.

## Discussion

### Spontaneous activity

The spontaneous activity of these neurons is quite low. Values (0.08 ± 0.06 Hz) are lower than those recorded in the ventroposterior medial thalamus [VPM, 14.2 Hz under halothane, 7.9 Hz under pentobarbital, [30,36]] and somatosensory cortex [1.1 Hz, [30]]. This is unlikely due to a general anesthesia-induced suppression of spontaneous activity, as we found many neurons with high rates of spontaneous activity in all layers of the colliculus. The low rate of spontaneous activity in vibrissa-responsive units suggests that this pathway is equipped to respond with high signal-to-noise ratios.

### Receptive fields

The receptive fields of collicular neurons are unique compared to those in other areas of the vibrissa-barrel pathway. Receptive fields are large (median = 14.5, 13.8 ± 0.1), with none of the cells responding only to a single vibrissa. Receptive fields in barrel cortex tend to be smaller, with 55% of neurons responding only to a single vibrissa [26]. The VPM nucleus of the thalamus contains neurons which respond to an average of three vibrissae, [± 1, [37]], receptive fields that are considerably smaller that those of collicular neurons. Collicular receptive fields are also larger than those in the second somatosensory cortex, previously characterized as having unusually large receptive fields [median = 9, [28]]. In the paralemniscal POm (posterior nucleus of the thalamus), where receptive fields are also large, usually only stimulation of many vibrissae can evoke a response [38,39]. By contrast, all collicular neurons were capable of responding to deflections of an individual vibrissa. A principal whisker could be identified based on response magnitude, while responses to adjacent vibrissae were also robust. The broad receptive fields we observed could be an artifact of anesthesia. However, anesthesia tends to sharpen vibrissa receptive fields, and not to broaden them [40,41].

### Angular preference

Collicular neurons are more selective to the angle of vibrissa deflection, compared to neurons in VPM or barrel cortex: 43 percent of collicular neurons have a selectivity index of 5 or greater, whereas only 31% of VPM cells and 16% of barrel cortex neurons have an equally high selectivity index [30]. The superior colliculus receives its main trigeminal input from the trigeminal sub-nucleus interpolaris (SpVi), and angular tuning of collicular neurons may reflect the properties of these input neurons, which contains a population of highly tuned neurons [42]. However, this tuning is not always consistent across the receptive field of collicular neurons (e.g., Fig. 3A). This may be a result of convergence from neurons in SpVi that are tuned to different directions.

### Response kinetics

Collicular neurons respond to whisker deflections at short latencies (5.9 ± 0.4 msec). These latencies are similar to those previously reported in VPM [6.4 ± 1.8 msec, [43]]), and are shorter than those in POm [27.4 ± 2.2 msec [22]]. Thus, consistent with anatomical findings (see Background), this suggests that collicular neurons are receiving input directly from the trigeminal nucleus, and are not being activated by cortical or thalamic loops.

Because the superior colliculus receives its main trigeminal input from the SpVi, it is considered part of the paralemniscal system [44]. However, the colliculus displays more robust responses than POm, which also receives direct input from SpVi. Responses in POm are characterized by their long latency (see above) and low magnitude [0.7 ± 0.1 spikes/stim, [22]], compared to collicular responses (1.9 ± 0.4 spikes/stim). Additionally, neurons in the paralemniscal system, including POm, typically respond poorly at increasing stimulation frequencies [38,45]. Collicular neurons, in comparison, respond to 15 Hz stimulation with 38% of the response magnitude evoked by 0.5 Hz stimulation (0.6 spikes/stim compared to 1.6 spikes/stim). However, POm responses are enhanced when inhibition from zona incerta to POm is suppressed [20,22]. It remains to be determined if the colliculus, which is reciprocally connected with zona incerta [19], is also regulated by inhibition from this structure.

Even when inhibition from zona incerta is intact, collicular responses are large (median = 1.2 spikes/stim), and similar to those seen at several levels of the lemniscal system: 0.99 spikes/stimulus in VPM [46] and 1.8 spikes/stimulus in barrel cortex [28]. The superior colliculus does receive some input from the trigeminal nucleus principalis [44,47], and these inputs could be driving these robust collicular responses.

The ratio of rapidly adapting to slowly adapting neurons decreases along the vibrissa-to-cortex neuroaxis. This reduction is greater in the paralemniscal system. The proportion of slowly adapting neurons in the lemniscal system is relatively large: 93% in the principal trigeminal nucleus [34], 37% in VPM, and 15% in barrel cortex [30]. The paralemniscal pathway begins with a smaller proportion of slowly adapting neurons, with 54% of the cells in SPVi [48] and only 3% of the neurons in the second somatosensory cortex [28]. In the superior colliculus, none of the neurons we recorded from were slowly adapting, lending further support to the assumption that the colliculus is part of the paralemniscal system.

OFF:ON response magnitude ratios in the superior colliculus (1.4 ± 0.5) are larger than those of excitatory barrel cortex neurons (0.55 ± 0.03, 29). Experimental and modeling studies indicate that smaller OFF:ON ratios in barrel cortex are a consequence of the dominance of inhibition in the layer IV damping circuit [31]. The larger OFF:ON ratios in the colliculus may reflect a lesser role for inhibition in this structure, at least under our experimental conditions. Whereas inhibition in barrel cortex may be important for spatiotemporal discrimination, lesser inhibition in the superior colliculus may optimize this structure for alerting and orienting functions.

Collicular neurons had decreased response magnitude and increased CV in response to higher frequency stimuli (Fig. 6F). Nevertheless, collicular neurons displayed precise phase locking at all frequencies tested, with low average coefficient of variance for the latency (from 0.04 at 0.5 Hz to 0.1 at 15 Hz). These values are much smaller than those reported in POm, which ranged from 0.58 to 0.82 over 0.5 to 11 Hz [38]. Thus, collicular neurons respond to high-frequency stimulation with more temporal precision than neurons in other parts of the paralemniscal system. The high response fidelity of superior colliculus neurons demonstrates that this structure is well equipped for accurate frequency discrimination.

The high OFF:ON ratios and robust, time-locked responses indicate that the superior colliculus is well suited for the accurate detection of a novel or relevant stimuli. Although collicular neurons entrain to repetitive stimuli, response probability decreases as the cells habituate (Fig. 6). This suggests that non-novel stimuli may result in reduced output from collicular circuits. These issues need to be addressed in unanesthetized, behaving animals.

## Conclusion

Several aspects of the neuronal response properties discussed here are consistent with superior colliculus involvement in orienting responses and alerting or attentive functions related to somatosensation in the rat. The low levels of spontaneous activity create the potential for high signal-to-noise levels both in collicular processing and in efferent signaling. The short latency, large magnitude, and reliability of the responses are behaviorally advantageous for rapid and consistent awareness of novel or relevant stimuli. The apparent minor role of inhibition, as evidenced in the large OFF:ON ratios, may also be optimized for alerting functions. The interconnections of the superior colliculus with many other structures involved in sensation (thalamus, cortex) and arousal (zona incerta) suggests this structure may play a role in regulating awareness of vital stimuli.

## Methods

### Surgical procedures

We performed experiments using 24 female Sprague-Dawley rats weighing 200 to 350 g. All procedures strictly adhered to institutional and federal guidelines. We maintained the rats within anesthetic stages III-3 to IV [40] with isoflurane (1.0 to 2.0%), administered through a tracheal tube. Body temperature was maintained at 37°C with a servo-controlled heating blanket. Following infusion of local anesthetics at surgical sites, we performed a craniotomy (1.5 mm diameter) over the cortex covering the superior colliculus.

### Recording

We obtained extracellular unit recordings with either quartz-insulated platinum electrodes or glass-insulated tungsten electrodes (both 2 to 4 MΩ). Electrodes were advanced perpendicular to the cortical surface, using either an Inchworm stepper motor (Burleigh, Fishers, NY), or a multi-electrode manipulator (Alpha Omega, Israel or Thomas Recording, Germany). We continuously stimulated vibrissae on the contralateral face during electrode penetrations to detect units with low or no spontaneous activity. Waveforms recorded from well-isolated units were digitized at 40 kHz through an AlphaLab data acquisition system (Alpha Omega). We isolated units off-line with a combination of threshold and waveform component analysis using Off-Line Sorter (Plexon, Dallas). We report data from 48 single units and 3 multiunit recordings; we combined data from multiunits and single units as these sets were indistinguishable.

Recording sites were marked with electrolytic lesions (5 to 10 μA for 20 sec). The animals were deeply anesthetized with sodium pentobarbital (60 mg/kg) and perfused transcardially with buffered saline followed by 4% buffered paraformaldehyde. Recording sites were identified in Nissl-stained coronal sections.

### Vibrissa stimulation

We determined the size of receptive fields by manually deflecting individual vibrissae. Two types of controlled vibrissa stimulation were used: A piezoelectric device that deflected a single vibrissa, or an air-puff stimulus that deflected several vibrissae. Vibrissae deflected individually were inserted into a small tube–approximately 10 mm from their base–attached to a computer-controlled piezoelectric stimulator that can be deflected in eight different directions (courtesy of Dr. D.J. Simons, Univ. Pittsburgh). We applied ramp-and-hold stimuli, either 200 ms or 30 msec in duration, having onset/offset velocity of 102 mm/s. The shorter stimulus duration was used to compare responses to different frequencies of repeated stimuli (see below). To reduce mechanical ringing, the trapezoid ramp-and-hold waveforms were filtered with a Bessel filter. The peak onset and offset velocity were measured as the slope of the linear portion of the deflection ramp. We calibrated the stimulator with a photodiode device.

The piezoelectric stimulator was used to compare responses both to different frequencies of stimulation and to different angles of deflection. In the frequency paradigm, all deflections were in the caudal direction at varying frequencies (0.5, 1, 2, 5, 8, 11 or 15 Hz). In the angular paradigm the vibrissa was deflected in eight different directions (in 45° increments). In these experiments, deflections were at 1 Hz with a pseudo random sequence for a total of 15 stimuli per deflection angle (120 deflections total).

Air-puff stimuli deflecting several vibrissae were delivered through a tube (0.5 mm diameter) by a Picospritzer™ (General Valve, Fairfield, NJ). We delivered air-puffs at 0.5, 1, 2, 5, 8, 11 or 20 Hz, with a pressure of 60 psi, resulting in vibrissa deflections of approximately 30°. The duration of the air-puff was 200 msec, except when testing the response to different frequency stimuli, when the air-puff duration was reduced to 30 msec (for 20 Hz stimuli) or 50 msec (for all other frequencies).

In our analyses of response kinetics, we included data on responses to stimulation at the lowest frequency tested for each neuron (0.5 Hz, 1 Hz, or 2 Hz). There were no significant differences in any response kinetic tested in response to these different frequencies.

### Data analysis

Time stamps of well-isolated units and of stimulus triggers were exported to Matlab (MathWorks, Natick, MA) for analyses using custom written software. Peristimulus time histograms (PSTHs, 0.2 msec bins) were constructed from these time stamps. For display purposes, PSTHs in the figures were constructed using 1 msec bins. Significant stimulus-evoked responses were defined as PSTH bins whose response magnitude significantly exceeded (99% confidence interval) spontaneous activity levels, computed from a baseline period preceding each stimulus. Response onset was defined as the occurrence of two consecutive post-stimulus bins (0.4 msec) displaying significant responses. Response offset was defined as 10 consecutive bins (5 msec) in which no significant response occurred. We used these criteria to objectively and consistently define significant response epochs, relative to baseline firing.

Statistical analyses were performed in SPSS and Microsoft Excel. Where appropriate, results are displayed using a boxplot to depict the median and distribution of the data (see Fig. 2D). Between-group statistical comparisons were assessed with the nonparametric Kolmogorov-Smirnov (K-S) test, because it is sensitive to any type of distributional differences (i.e., central tendency, variability, skewness, and kurtosis) and makes no assumptions regarding normality or equivalence of variance. Tests for within-group comparisons are specified in the text. Data are presented as median or mean ± standard error.

## Authors' contributions

MEH participated in designing the study, carried out electrophysiological experiments and performed the analysis. AK conceived and directed the project. Both authors drafted the manuscript and have approved the final manuscript.
